# Validation of a magnetic resonance imaging guided stereotactic access to the ovine brainstem

**DOI:** 10.1186/s12917-014-0216-5

**Published:** 2014-09-22

**Authors:** Anne Staudacher, Anna Oevermann, Michael H Stoffel, Daniela Gorgas

**Affiliations:** Division of Clinical Radiology, Department of Clinical Veterinary Medicine, Vetsuisse-Faculty, University of Berne, Längassstrasse 128, CH 3012 Berne, Switzerland; Divison of Neurological Sciences, Department of Clinical Research and Veterinary Public Health, Vetsuisse-Faculty, University of Berne, 3012 Berne, Switzerland; Institute of Veterinary Anatomy, Vetsuisse-Faculty, University of Berne, 3012 Berne, Switzerland

**Keywords:** Sheep, Brainstem, Stereotaxy, Transcerebellar route, Large animal model

## Abstract

**Background:**

Anatomical differences between humans and domestic mammals preclude the use of reported stereotactic approaches to the brainstem in animals. In animals, brainstem biopsies are required both for histopathological diagnosis of neurological disorders and for research purposes. Sheep are used as a translational model for various types of brain disease and therefore a species-specific approach needs to be developed. The aim of the present study was to establish a minimally invasive, accurate and reproducible stereotactic approach to the brainstem of sheep, using the magnetic resonance imaging guided Brainsight^TM^ frameless stereotactic system.

**Results:**

A transoccipital transcerebellar approach with an entry point in the occipital bone above the vermis between the transverse sinus and the external occipital protuberance was chosen. This approach provided access to the target site in all heads. The overall mean needle placement error was 1.85 ± 1.22 mm.

**Conclusions:**

The developed transoccipital transcerebellar route is short, provides accurate access to the ovine caudal cranial fossa and is a promising approach to be further assessed in live animals.

## Background

Currently, stereotactic brain biopsy is the least invasive method to obtain brain tissue [1-4], especially from lesions that are deep-seated [2,5-7] or located in vitally important intracranial regions such as the brainstem [5,6,8-10]. Three different approaches to the brainstem have been described in people: the transtentorial, transfrontal and suboccipital transcerebellar route [11-13]. Since traversing the tentorium might cause pain and/or hemorrhage at the pial surfaces of the cerebellum or mesencephalon [9,11] and potentially damage vital blood vessels and cranial nerve nuclei, the transtentorial route is no longer used [11]. Depending on the location of the lesions, either a transfrontal [4,9,14-16] or a suboccipital transcerebellar [15,17-20] approach is used. For midbrain lesions, targets located along the midline of the brainstem and for lesions in the caudal part of the medulla oblongata, a transfrontal coronal route is used, whereas for lesions located laterally or within the cerebellum, cerebellar peduncles and pons, a suboccipital transcerebellar route through the middle cerebellar peduncles is preferred [21-24].

In animals, brainstem biopsies are required both for histopathological diagnosis of neurological disorders and for research purposes. Sheep are used as a translational model for various types of brain diseases in humans [25-27] and brainstem biopsies are necessary to investigate the neuropathogenesis of listeric rhombencephalitis, the most frequent central nervous system disease of ruminants. However, the anatomical differences between species do not allow methods developed in humans to be transferred directly to the sheep.

**Figure 1:**
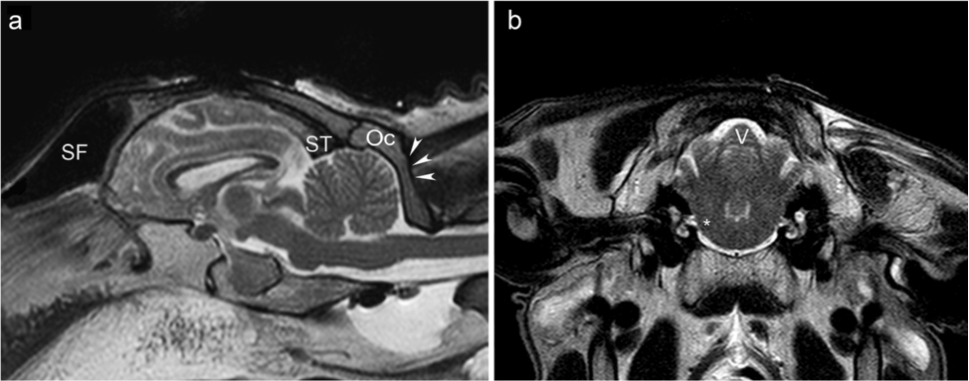
**Sagittal and transverse T2 weighted magnetic resonance images of the head of a sheep.** The sagittal image **(a)** shows the large frontal sinus (SF) rostral to the forebrain, and the transverse sinus (ST) as rostral limitation of the transcerebellar approach. The attachment of the neck musculature at the occipital squama (arrow heads) defines the caudal limitation of the approach leaving a small area of the occipital bone (OC) as entry point for the trajectory. The transverse image **(b)** shows the vermis of the cerebellum (V) and the brainstem at the level of the emergence of the facial nerve (*). Note the different nomenclature for the anatomical planes in veterinary medicine.

The tetrapod gait of domestic mammals entails a horizontal brain axis where the brainstem is not situated underneath, but caudal to the forebrain [28], which precludes the use of a transfrontal route to the brainstem (Figure 1). Furthermore, the frontal sinuses of most domestic mammals are larger than in humans and cover a greater portion of the rostral brain surface [29]. Traversing the frontal sinuses is inadvisable [30] because of increased morbidity due to intrasinusoidal bleeding/epistaxis [31], wound infections [32] or subcutaneous emphysema [33]. It also prolongs the trajectory and poses the risk of instrument deviation [34] at the compact bone [23] between the sinus and the dura where the needle cannot be controlled visually.

The intraparenchymal transcerebellar approach to the brainstem is inadequate in domestic mammals because of the lateral position of the cerebellar peduncles [28]. Moreover, the caudal contour of the cerebellum is covered by the squama occipitalis, and the attachment of nuchal muscles precludes a suboccipital transcerebellar approach (Figure 1).

Consequently, stereotactic approaches to the brainstem used in human medicine cannot be applied to sheep. In veterinary medicine, brain targets located within the caudal cranial fossa were rarely addressed stereotactically [35,36], but the employed approach was not mentioned. In the present study an applicable transoccipital transcerebellar magnetic resonance imaging guided stereotactic approach to the brainstem of sheep is described and its target accuracy was determined using the modified Brainsight^TM^ stereotactic system.

## Results

**Figure 2:**
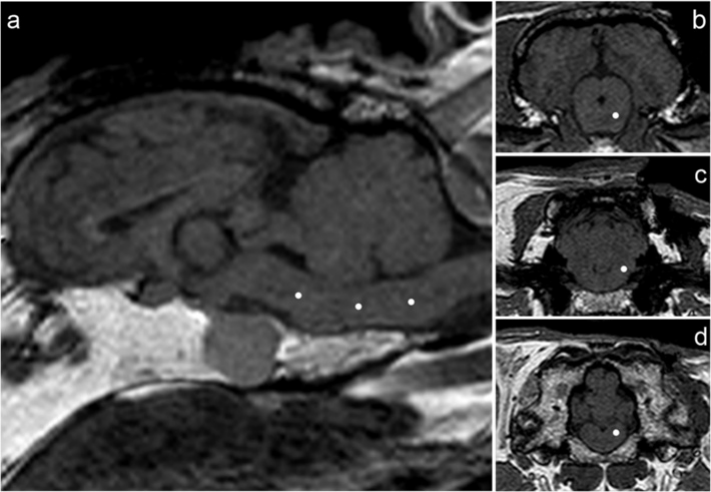
**T1 weighted 3D gradient echo sequences for target planning.** The targets (white dots) were planned within the brainstem at three different locations **(a)** at the level of the mesencephalon **(b)**, the facial nerve **(c)**, and the obex **(d)**.

**Figure 3:**
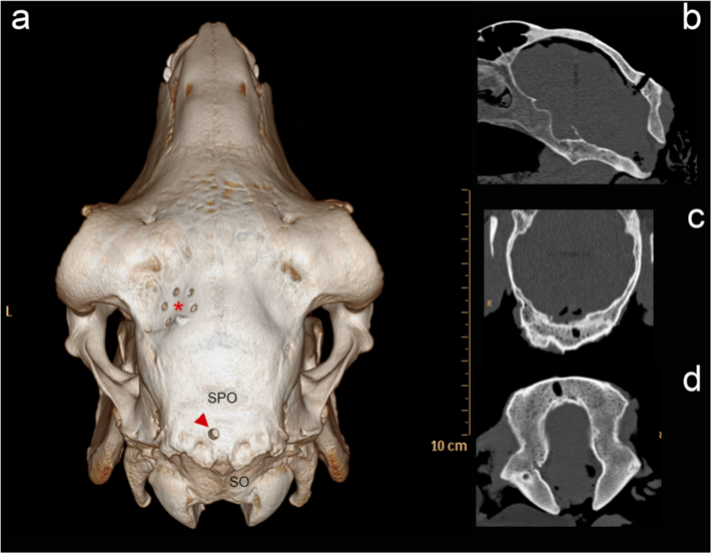
**3D reconstruction and multiplanar reconstructions of computed tomographic images of an ovine skull.** The 3 D model **(a)** shows the drill hole (red arrowhead) for targeting the emergence of the right facial nerve, which is located slightly to the left of the middle between the sutura parietooccipitalis (SPO) and the nuchal crest. A red asterix is placed in the middle of the burr holes for the fiducial marker post. The multiplanar reconstructions in the transverse **(b)**, dorsal **(c)** and sagittal **(d)** plane demonstrate the burr hole in the different orientations. SPO: Sutura parietooccipitalis; SO: Squama occipitalis.

The transoccipital transcerebellar approach with its entry point in the occipital bone above the vermis between the transverse sinus and the external occipital protuberance allowed access to the target site in all of the eighteen cadaver heads (Figures 2, 3). Attachment of the fiducial marker post, acquisition of both sets of magnetic resonance images (MRI), planning of the trajectory and establishment of the target coordinates and the stereotactic injection of the contrast medium took 30, 40, 30 and 45 minutes, respectively.

The mean needle placement error for the midbrain (n = 6), pons (n = 6) and obex (n = 6) targets was 1.77 ± 1.47, 2.48 ± 1.16 and 1.28 ± 0.83 mm, respectively. The overall mean needle placement error for all target sites (n = 18) was 1.85 ± 1.22 mm. The mean target depth for the midbrain, the pons and the obex targets was 36.9 ± 2.36, 33.18 ± 0.82 and 29.6 ± 1.50 mm, respectively. There was no statistically significant relationship between needle placement error and target depth (*P* = 0.28).

Macroscopic evaluation revealed that toluidine stains were visible in the region of sixteen of the eighteen targeted sites. In two brains, a blue stained margin was observed at the edge of the obex. The position of the toluidine stain was at the target site in thirteen sheep heads. The dot was displaced in three brainstems. It was found rostrally to one target in the left midbrain, medially to one target in the right pons and lateroventrally to a destination in the left pons.

## Discussion

The stereotactic transoccipital transcerebellar approach to the ovine brainstem used in this study was applicable in all the heads used. The overall mean needle placement error in the brainstem of 1.85 ± 1.22 mm is comparable to previous results of the magnetic resonance imaging guided Brainsight^TM^ stereotactic system in targets in the canine rostral and middle cranial fossa (mean needle placement error of 1.79 ± 0.87 mm) [36]. Reported mean needle placement errors of CT-guided stereotactic systems used in veterinary medicine are larger for targets within the rostral, middle and/or caudal cranial fossa [30,31,37], and only slightly smaller (1.7 ± 1.6 mm) for targets exclusively located in the rostral cranial fossa [33].

The obtained error was also smaller in comparison with the *in vivo* accuracy of frameless MRI guided stereotactic brainstem biopsy sampling in human medicine [7,38]. Frameless systems have replaced the stereotactic frame with a method of registration that relies on anatomic landmarks - such as nose, eyes and ears - or artificial markers, called ‘fiducials’. The latter are attached to the patient’s head before the brain scan, and a three-dimensional digitizer matches them to the corresponding points in the image. Frameless systems provide a wide range of motion for the instrument guidance arm [39].

We consider the transcerebellar approach with its entry point in the occipital bone above the vermis between the transverse sinus and the external occipital protuberance to be the only one to be safely applied to the whole ovine brainstem (Figure 1). The brain surface through which a trajectory to the brainstem can be placed is rostroventrally confined by the large frontal sinuses [30-33]. As in people, the trajectory should avoid the membranous tentorium of the cerebellum [4]. Therefore, the transfrontal approach, which permits access to all divisions of the brainstem [4,9,16] has limited value in domestic mammals and the transcerebellar route is more promising. However, a transcerebellar trajectory may not enter through the squama occipitalis, the part of the occipital bone situated caudally to the cerebellum, because the required shallow angle between bone and drill bit would result in slippage of the drill bit [36]. Furthermore, the occipital squama of sheep is covered by a considerable amount of muscles, whose dissection is known to cause massive postoperative wound pain in people [7,11,40]. Consequently, the entry point has to be placed rostral to or on the external occipital protuberance (Figures 1, 3).

Attention must be paid to the vasculature, notably the transverse sinus [7,12,18,19,41], the dorsal cerebellar veins and dorsal rami of the caudal cerebellar arteries between the vermis and the cerebellar hemispheres [28], which limit the area allowing the caudal cranial fossa to be accessed cranially and laterally, respectively.

**Figure 4:**
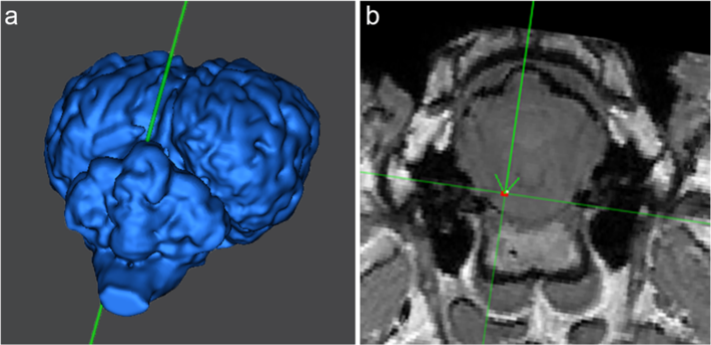
**3D reconstruction (a) and T1 weighted transverse MRI (Brainsight** ^**TM**^ **) (b) for trajectory planning for the target at the level of the facial nerve.** Note the entry point above the cerebellar vermis and the intraparenchymal course of the trajectory.

Usually, the suboccipital transcerebellar trajectory to the brainstem in people is placed through one of the middle cerebellar peduncles [13,16,17,19,20,23,24,40-43]. This approach, however, is not feasible in domestic mammals because of the more lateral position of these structures [28]. Therefore an access route through the zone of greatest contiguity of the brainstem to the cerebellum, which spares the fourth ventricle [7,44] and is used as an alternative approach in people [18,45], was applied in the present study (Figure 4).

Using this approach, there is no lateral restriction to the described trajectory. Another important advantage is the shortness of the trajectory [12,13,16,17,19,21,23,40], which results in minimized tissue trauma as well as increased accuracy [17,46].

Disadvantages of the human suboccipital transcerebellar compared to the transfrontal approach such as the need for general anesthesia [9], prone positioning [23,40] and considerable muscle dissection [7,11,40] are irrelevant in veterinary medicine: all the patients need to be subjected to general anesthesia, thus rendering participation in an intraoperative neurological examination impossible [9,11]. The tetrapod anatomy suggests prone positioning during the MRI examination and the interventional procedure, which is a comfortable operating position for the surgeon [19] and complies with the physiological patient composure so that brain shift is a minor concern [23]. In this cadaver head study, loss of CSF and elasticity of the brain parenchyma as well as loss of continuity of the brainstem with the spinal cord may have induced brain shift [23,40,47]. On the other hand, lack of parenchymal excursions in temporal synchrony with systole [47] could have led to underestimation of targeting error, but this impact can only be assessed in a clinical setting.

Other causes of needle placement error might also have occurred during target registration, fiducial registration, and target positioning [48]. In order to minimize these errors, a rigid head fixation was ensured and the registration was checked after each step that could have caused slippage of the head in the clamp or movement of the freeguide arm [46]. In accordance with published recommendations, the fiducial markers implanted into the bone were carefully monitored throughout the whole procedure by the same previously trained person [32,34,46].

As judged from macroscopic evaluation, the toluidine dot was displaced in three specimens. In all three specimens, the actual needle placement error was higher than the mean needle placement error (4.35 mm, 3.15 mm and 2.01 mm). Nevertheless, in some objects with a large needle placement error (4 mm, 3.23 mm), the toluidine stain was not judged to be off target macroscopically, possibly due to the fact that an error with a large deviation in a single plane is more striking than an error with a small deviation in all three planes. In two brains, in which no toluidine stain but a blue margin of contrast medium at the edge of the obex was detected, the contrast medium presumably leaked when the injection needle pierced the meninges, a known drawback of stereotactic drug delivery [49,50], which is probably of minor importance in stereotactic brain biopsy sampling. Access to the rostral mesencephalon is limited by the tentorium and the external occipital protuberance, which prohibits more caudal tilting of the trajectory. Therefore, in our experience, the crura cerebri are the most rostral area, which can be accessed through the cerebellum. The caudal restriction of the transcerebellar approach is given by the caudal border of the cerebellum, making the obex the most caudal area which can be reached. Additionally the transverse sinus prevents further rostral tilting of the trajectory.

Consequently, the aforementioned transoccipital transcerebellar approach gives access to all structures within the ovine caudal cranial fossa including the cerebellum, cerebellar peduncles and lateral regions of the brainstem.

Intracranial hemorrhage and postoperative neurological deficits are a major concern in human stereotactic brainstem biopsy [13,18,43,45] and could also arise in sheep. Although the herein developed approach to the ovine brainstem avoids the transverse sinus, the dorsal cerebellar veins, the dorsal rami of the caudal cerebellar arteries between the vermis and the cerebellar hemispheres and cranial nerves within the brainstem, the risk of bleeding from smaller vessels as well as the occurrence of neurological deficits remains to be systematically assessed in living sheep.

In contrast to sheep, the canine skull has more prominent bony crests in the occipital area. Depending on dog breed and size, this prohibits an entry point in the midline of the occipital bone. However, the anatomy of the ovine, canine and feline head is otherwise similar so that the herein described approach to the brainstem can theoretically be translated to dogs and cats [29]. The authors have already employed a transoccipital transcerebellar approach in canine cadavers.

## Conclusions

The study proved a stereotactic transoccipital transcerebellar approach to be suitable to access targets along the whole axis of the ovine brainstem with good accuracy and is currently used to sample the brainstem in live sheep. Possibly associated complication rates and the application of the access in other species can now be assessed in further studies.

## Methods

Eighteen one-year-old healthy sheep of different breeds (Swiss White Alpine Sheep (n = 3), Black- and Brownheaded Mutton (n = 3 and n = 12, respectively) were slaughtered in the context of food production. The study was performed in agreement with the local ethic regulations (Swiss Veterinary Service, Office of Agriculture and Nature (LANAT)). The heads were disarticulated at the atlantooccipital junction and stored at + 6 °C within 48 hours upon arrival until use. Mean weight of the heads was 2.96 ± 0.91 kg (1.92-4.62 kg). The stereotactic procedures were performed using the Brainsight^TM^ frameless stereotactic system (Rogue Research Inc., Montreal, Canada) [36] with a modified bone implanted fiducial marker system [46,48,51,52]. After clipping of the coat and placement of the sheep head in the C-clamp, the fiducial marker post was fixed on the frontal bones caudal to one of the zygomatic processes to ensure that the registration markers were in different planes and not obstructing the surgical site. Hence, the distance from the centroid of all fiducial markers to the planned target was small [46,48,52] and suitable bone thickness for post fixation was ensured. The implant post was attached with at least three 8 mm-ceramic screws. A fiducial array hub with five fiducial markers was screwed onto the post.

The cadaver head was scanned in a 1.0 Tesla MRI system (Philips Panorama HFO, Philips System, Best, The Netherlands) in prone position using a head coil. A T1-weighted gradient echo 3D sequence was acquired using the following parameters: TR = 25 ms, TE = 6.9 ms, flip angle = 30°, Number of Signal Averages = 2, slice thickness = 1.8 mm without interslice gap. The field of view was adjusted according to skull size and position of fiducial markers.

Three different target sites within the brainstem were determined: ventral part of the midbrain directly adjacent to the pons, pons at the level of the emergence of the facial nerve or obex, on the right or left side, respectively (Figure 2). This resulted in three cadaver heads per target site. For each target, coordinates (X, Y, Z) were read out using the Brainsight^TM^ neuronavigation software. The trajectory for all the targets was planned via a transoccipital transcerebellar access (Figures 3, 4).

Following acquisition of the MRI, the sheep head with the fiducial marker system was again fixed in the surgical headclamp in prone position using four skull screws. The open side of the surgical C-clamp was directed caudally. The two skull screws at the caudal end of the clamp were placed in the temporal fossa. The rostral skull screws made contact with the nasal bone.

The subject to image-registration was performed [36] with the Polaris® optical position sensor placed rostrally to the cadaver head and checked directly before and after drilling and after removal of the manual ruler guide from the instrument sleeve. The neuronavigation pointer was inserted into the instrument sleeve of the articulated arm. The entry point was kept as perpendicular to the skull surface as possible to prevent slippage of the drill bit. Tight locking of the articulated arm [46,52], correct use of the stabilization pin and good contact between the drill guide tube and the skull [46] were ensured. Furthermore the instrument receptacle of the articulated arm was fixed manually during the drilling of a 5 mm burr hole to prevent residual movement of the assembly.

The neuronavigation pointer was inserted in the instrument sleeve and lowered down to a zeroing platform. The distance from the zeroing platform to target was determined by the software [36]. The manual ruler guide with a 10 μl syringe and attached 26-gauge, 6-inches needle (Hamilton Company, Reno NV/Bonaduz, Switzerland) filled with the contrast solution and straightened by a guiding cannula was then placed in the instrument sleeve of the articulated arm. The needle was lowered manually to the zeroing platform, the manual ruler guide was set to zero and after removal of the platform the needle was lowered to target.

Ten minutes after the needle reached the predetermined target depth, 0.5 μl of the contrast solution was injected. This contrast solution was made by adding 0.4 ml of gadodiamide (Omniscan®, GE Healthcare Inc., Glattbrugg, Switzerland) and 0.05 g of toluidine blue to 100 ml of 0.9% NaCl. The needle was kept in place for 5 min after the injection to prevent contrast medium leakage along the needle track. Immediately following the contrast injection, a second MRI study was performed using the same parameters as before. These images were uploaded and saved to the neuronavigation computer later on [36].

**Figure 5:**
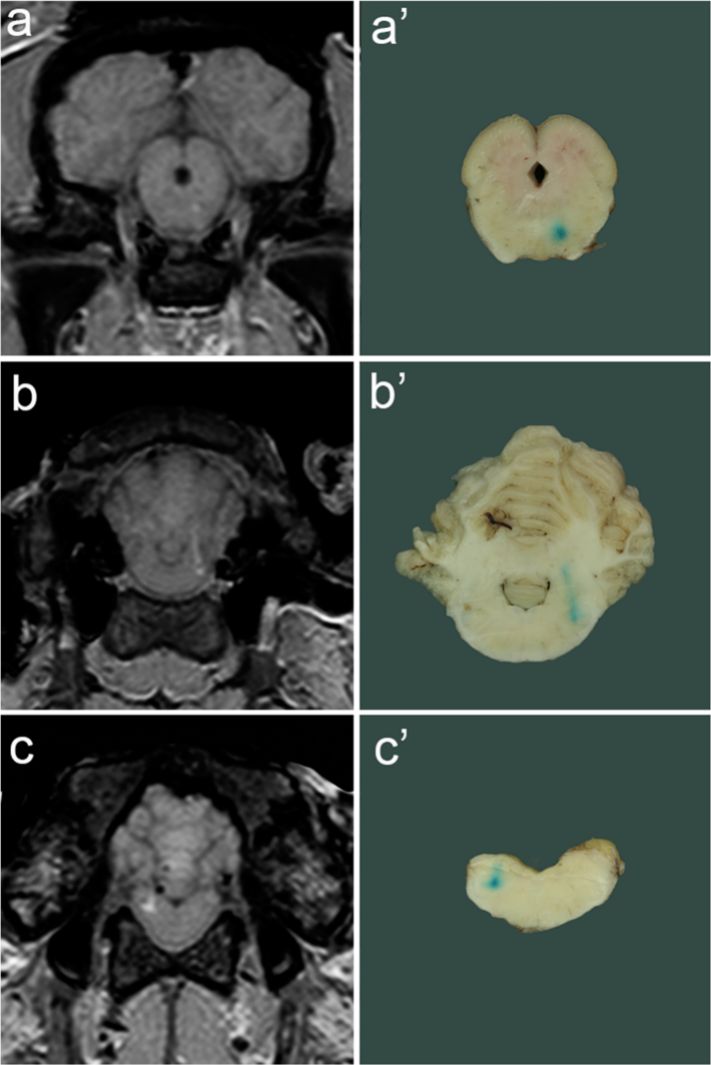
**Magnetic resonance images and macroscopic samples for target comparison.** Needle placement error was calculated comparing the coordinates of the planned target site and the contrast bloom on magnetic resonance images **(a, b, c)** and assessed subjectively comparing the location of the toluidine stain on macroscopic samples with the planned target sites **(a’, b’, c’)**.

Thereafter the brain was unhinged and fixed in formalin for eight weeks. Subsequently, all the brains were sliced and the contrast stains were judged to be present or absent by a neuropathologist (A.O.). The position of the toluidine dot was assessed subjectively to be at or off the targeted site (Figure 5).

To determine the mean needle placement error, the postoperative MR images were registered to the preoperative ones using the Image Feature of the Brainsight^TM^ neuronavigation software. The preoperative MRI served as the reference so that a common coordinate space was used between the preoperative and postoperative MRI. The planned injection site represented target A and the center of the gadodiamide deposition target A’ [36]. Coordinates of A’ (X’, Y’, Z’) were read off. In two cases, the contrast bloom was not clearly visible but a gas bubble was depicted at the tip of the needle track on the MRI. The center of these gas bubbles was taken as the target. The precision of the system in bringing the needle to target (needle placement error) was calculated for each target site using the formula: Error = √[(X-X’)^2^ + (Y-Y’)^2^ + (Z-Z’)^2^]. Mean needle placement error and standard deviation (SD) were then assessed based on all the target sites. Linear regression was used to evaluate the relationship between needle placement error and target depth in the brain. *P* value < 0.05 was considered significant [36].
